# The Awareness of Rare Diseases Among Medical Students and Practicing Physicians in the Republic of Kazakhstan. An Exploratory Study

**DOI:** 10.3389/fpubh.2022.872648

**Published:** 2022-04-08

**Authors:** Dariusz Walkowiak, Kamila Bokayeva, Alua Miraleyeva, Jan Domaradzki

**Affiliations:** ^1^Department of Organization and Management in Health Care, Poznan University of Medical Sciences, Poznań, Poland; ^2^Department of Pediatric Gastroenterology and Metabolic Diseases, Poznan University of Medical Sciences, Poznań, Poland; ^3^Department of Psychology, West Kazakhstan Marat Ospanov Medical University, Aktobe, Kazakhstan; ^4^Department of Social Sciences and Humanities, Poznan University of Medical Sciences, Poznań, Poland

**Keywords:** rare diseases, physicians, medical students, healthcare, Kazakhstan, medical education

## Abstract

Although national plans or strategies for rare diseases (RDs) have been implemented in many jurisdictions research show that one of the main barriers RD patients face during medical encounter is medical professionals' low level of knowledge and experience on the diagnosis, treatment and rehabilitation of RD patients. Consequently, there is a need to increase the standards of medical education in the field of RDs and to revise the undergraduate and postgraduate training programs. However, while studies on medical education in the field of RDs has been conducted in various countries across the both Americas, Asia or the European Union, still little is known about the awareness of RDs among healthcare professionals in the Republic of Kazakhstan. Thus, we conducted a survey among 207 medical students and 101 medical doctors from the West Kazakhstan Marat Ospanov Medical University, Aktobe, Kazakhstan. The study was conducted between March and May 2021. The questionnaire assessed their knowledge about the number, examples, etiology and estimated frequency of RDs. It also evaluated respondents self-assessment of competence in RDs. Although the majority of respondents agreed that RDs constitute a serious public health issue both medical students and medical doctors showed insufficient knowledge on the etiology, epidemiology and prevalence of RDs, and many had problems with separating RDs from more common disorders. Moreover, they also lacked knowledge about and the central register of RD patients and reimbursement of orphan drugs in Kazakhstan. Finally, while almost half respondents declared having had classes about RDs during their studies most perceived their knowledge about RDs as insufficient or poor and felt unprepared for caring for RD patients. Additionally, although majority of respondents in both groups believed that all physicians, regardless of their specialization, should possess knowledge on RDs many respondents did not look for such information at all.

## Introduction

Ever since the Orphan Drug Act was passed in the United States in 1983 rare diseases (RDs) have been widely recognized as an urgent medical, legal, economic, social and public health problem (1). Consequently, countries around the world have developed many areas of health policy in the field of RDs, including the classification and codification of RDs and ICD-10 revision, improving prevention and recommendations in funding and the reimbursement of orphan drugs and the creation of national registrations of RD patients. Moreover, many jurisdictions have created or implemented national plans or strategies for RDs (2–12). However, although previous studies highlight how RDs have become a policy priority in various countries across the both Americas, Asia or the European Union (2, 6, 12–15), still little is known about health policy toward RDs in countries from Central Asia, including the Republic of Kazakhstan (RoK) (2, 12, 13, 15).

Nevertheless, due to the need to develop new solutions in the field of RDs during past few years the issue of rare diseases is attracting more and more attention in Kazakhstan. Consequently, in accord with the Regulation Order of the Ministry of Healthcare of the RoK a List of Orphan Drugs has been registered in 2009 (13) and in 2016-2017 regional rare disease coordinators were appointed and trained (16, 17). Their mission is to monitor the situation in each region, identify new patients with RDs, enter them into a database, and assist such patients in their needs (17). Additionally, there are media coverage of events, conferences, seminars, discussions and meetings of health professionals and higher-ups, websites created. For example, Sanofi Genzyme has launched a first-of-its-kind app in Kazakhstan called the Rare Disease Guide. It is a practical guide for health professionals for the early diagnosis and management of lysosomal accumulation diseases (18). What is also important is that between 2016 to 2019 five PhD theses on RDs were defended in the RoK (19).

Moreover, in 2014 various patient organizations from Kazakhstan joined celebration of Rare Disease Day^1^, and in November 2020 the Association of Assistance to Patients with Orphan Diseases was organized in Kazakhstan. Its mission is to provide timely diagnosis, treatment and rehabilitation of patients with rare pathologies, as well as the organization of charitable assistance and social support.

Furthermore, a Roadmap for the implementation of new standards for diagnosis and treatment of RDs in children in the RoK for 2019-2020 was implemented. It aimed at developing methodological recommendations on the provision of medical care, revising and developing new clinical protocols, improving the laboratory service, monitoring the provision of patients with the necessary medicines, medical devices and medical nutrition, improving prevention and organizational measures, increasing staff capacity and conducting information and awareness-raising activities for the population (20). Presently, a Roadmap for improving the provision of comprehensive care for children with disabilities in the RoK for 2021-2023 is being implemented. It includes two main tasks: (1) expanding the list of medicines and medical devices for outpatient provision of children for all types of diseases, including rare diseases, and (2) training Primary Healthcare (PHC) physicians in diagnosis and treatment of ten specific rare diseases to create a multidisciplinary team in PHC medical institutions (21).

It is also worth noting that according to the Rules for development and revision of clinical protocols, the classification of a disease (condition) as a socially significant disease and/or rare disease is one of the main indications for prioritizing topics for development and revision of protocols (22).

However, although rare disease scene in the RoK has changed significantly, RD community in the country is still facing a number of challenges and unresolved problems which seriously halt the rate of progress and threaten the continued advancement of diagnostics, treatment and care for people with RDs. For example, the Scientific Center of Pediatrics and Pediatric Surgery has been aiming to establish a national register of RD patients in Kazakhstan, but it is still under the discussion (17). Consequently, while the Minister of Healthcare estimates the prevalence of RDs in Kazakhstan as 1 case per 2,000 (23) there are no official statistics on rare diseases in the country. At the same time, in the June 2021 the Head of the Department of Drug Provision and Standardization of the Ministry of Healthcare (MoH) declared that there were 46 362 RDs patients registered for follow-up in an Electronic Register of Dispensary Patients, of whom 71%, i.e., 32 936 were aged 18 or over, and 13 426 were children (29%) (24). Thus, while there is some progress in diagnosis of RD patients in RoK, this relatively low number of RD patients registered in the country results from both lack of awareness and knowledge on RDs among healthcare professionals and lack of appropriate coding systems, as Kazakhstan still does not relay on Orphacodes that can facilitate the classification and coding of RDs.

Simultaneously, in regards to newborn screening used to identify and effectively treat certain RDs at an early stage and to prevent irreversible damage, Kazakhstan only screens for phenylketonuria and congenital hypothyroidism (12). Moreover, although treatment of RDs is covered with the national healthcare budget no special reimbursement rules exist for orphan medicinal products (OMPs). However, OMP funding needs to be applied for by the regions, after which budget is granted by the State, based on individual patient characteristics (e.g., body mass/dosing). Moreover, although all medical interventions are supervised by the MoH no specific health technology assessment (HTA) process for OMPs exist in Kazakhstan (12, 25).

RDs patients in Kazakhstan also face problems with access to diagnosis and treatment which include a lack of quality diagnostics in the regions for certain types of RDs. Problems exist both at the initial stage of disease diagnosis and in the process of dynamic monitoring and treatment of RD patients. In addition, there is a need to improve the register to include all patient data, drugs, doses and dosages, to reflect continuity between services, to monitor the patient's condition during relocation and transfer to the Republican Medical Organization. The main problems of drug provision are: lack of registration of drugs in Kazakhstan; lack of a set ceiling price for procurement of drugs, interruptions in supply from “SK-Pharmacia” LLC (unified distributor, provides medicines to healthcare organizations and the population of the country under the Guaranteed Volume of Free Medical Care); insufficient work of Health Authorities to provide drugs from the local budget (12).

At the same time, it should be stressed that while RDs constitute a serious problem for patients and their families they also affect physicians and the healthcare system in general. While both the government and medical authorities stress that one of the most urgent areas in the health policy toward RDs is improving the medical education of healthcare students and professionals in RoK, still many healthcare professionals, including physicians, lack knowledge about RDs and are not prepared for caring for RD patients. The scarcity of knowledge, guidelines, and training on RDs of healthcare practitioners, seriously impede the diagnosis process, access to healthcare facilities and treatment options and management of such diseases. Consequently, RD patients themselves complain over the endless “diagnostic and therapeutic odyssey” (26, 27) and stress that it hampers timely diagnosis and treatment of patients suffering from a rare disease, especially when RD patients experience more common symptoms. This in turn results in the delays in referring patients for treatment, negatively affects their health, reduces patients' quality of life, and increases healthcare costs.

Thus, this study aims to assess the awareness of RDs among medical students and practicing physicians in the Republic of Kazakhstan (RoK).

## Materials and Methods

The study was conducted between March 2021 and May 2021 among students and medical doctors taking their specialization courses and medical doctors working at the West Kazakhstan Marat Ospanov Medical University, Aktobe, Kazakhstan. A previously developed questionnaire was used (28, 29), with which we had earlier tested the knowledge of Polish students and physicians. The questionnaire, which followed the guidelines of the European Statistical System (30), was translated into Russian, one of the two official languages of the Republic of Kazakhstan, and adapted to the Kazakh conditions. On the basis of the results of an online focus group, a working team (consisting of four general practitioners and one sociologist) decided which RD-related issues will be dealt with. Next, a provisional questionnaire was assessed by two external reviewers: one physician and one sociologist. Afterwards, our questionnaire was pre-tested by four other physicians using an online platform, which led to the reformulation of three questions. The final version of the questionnaire was again evaluated by two other external reviewers of the same specialties. The ethics approval and research governance approval were also obtained from the West Kazakhstan Marat Ospanov Medical University (Conclusion *N*o 6, protocol *N*o 2 of 02/18/2021). After the acceptance of the final version of the questionnaire, the survey was made available online. When recruiting doctors, invitations were sent to them via social media. In this group, the response rate was 100%. In the case of students, contact was made through group leaders, who were asked to provide their fellow students with a link to the questionnaire. Assuming that all students received this link, the response rate was 46%. However, it was most likely higher, since due to the fact that we have guaranteed our respondents full anonymity of the survey, we do not have any tool to verify the fact if a specific group of students has actually shared the link.

The questionnaire consisted of three sections. The first group of questions comprised the definition, etiology and estimated prevalence of RDs worldwide and in Kazakhstan. In this part of the questionnaire respondents were also asked to separate RDs from more common disorders from a list comprising 29 diseases. The second section addressed physicians' education about RDs and their self-assessment of their knowledge and competence in the field of these diseases. The last section referred to physicians' demographic data. The questionnaire consisted of 26 questions, of which we eventually used 25.

The data collected in the questionnaires were verified and checked for completeness, quality and consistency. Then they were coded and exported into the statistical packages JASP (Version 0.15.0.0). The results were presented as descriptive statistics. A Likelihood Ratio Chi-square was used to assess differences in the distribution of answers among the groups. A 5% level of significance was used for all hypothesis tests.

## Results

Our study group included 308 subjects, 207 (67.2%) of whom were students and 101 (32.8%) physicians (Table 1). Women predominated among both physicians (89.1%) and students (74.9%). In the group of students 152 (73.4%) were in their 5th year of study, while 55 (26.6%) were in their intern years. In the group of physicians, 40 (39.6%) were residents, and 61 (60.4%) professionally active physicians working at the university. Moreover 50.7% of the students and 37.6% of physicians have not met anyone suffering from RDs. Simultaneously, in both groups very few respondents declared having a family member suffering from such disease (4.8 and 3% respectively).

**Table 1:** Socio-demographic characteristics of respondents.

**Characteristics**	**N (%)**	
	**MD *n* = 101**	**Students *n* = 207**
Year of study		
5th		152 (73.4)
Interns 1^st^ year		28 (13.5)
Interns 2^nd^ year		27 (13.1)
Years of professional experience		
Residents 1^st^ year	16 (15.8)	
Residents 2^nd^ year	24 (23.8)	
<5	6 (5.9)	
6–10	4 (4)	
11–15	7 (6.9)	
16–20	10 (9.9)	
More than 20	34 (33.7)	
Gender		
Female	90 (89.1)	155 (74.9)
Male	11 (10.9)	52 (25.1)
Have you ever met a person suffering from RD		
Yes	55 (54.5)	94 (45.4)
No	38 (37.6)	105 (50.7)
I do not know	8 (7.9)	8 (3.9)
Is anyone in your family suffering from RD?		
Yes	3 (3)	10 (4.8)
No	93 (92.1)	194 (93.7)
I do not know	5 (4.9)	3 (1.5)

The majority of respondents were acquainted with the term ‘rare diseases’, which was known to 96% of physicians and 97.6% of students (Table 2). However, only 4.9% of physicians knew the frequency of the prevalence of RDs, whereas 4.9% correctly estimated the number of RDs. In the student group the results were equally poor, with 5.8% of students who knew the prevalence of RDs and 7.3% who correctly estimated the number of RDs. Similarly, a low number of respondents in both groups knew that RDs affect mostly children (17.8% of physicians and 14% of students). Moreover, both physicians and students had problems with estimating both the number of RD patients worldwide (1 and 4.4% respectively) and in Kazakhstan (2 and 2.4% respectively). Finally, while in both groups over 50% of respondents knew the most common cause of RDs, few were aware that the vast majority is of genetic character (physicians: 15.8%; students 16.9%).

**Table 2:** Respondents' knowledge about rare diseases.

**Characteristics**	***N*** **(%)**	
	**MD *n* = 101**	**Students *n* = 207**
Have you ever heard the term ‘rare diseases’?		
Yes	97 (96)	202 (97.6)
No	4 (4)	5 (2.4)
A rare disease is the one that affects less than:		
1 person in 1,000	5 (4.9)	24 (11.6)
**1 person in 2,000**	**5 (4.9)**	**12 (5.8)**
1 person in 3,000	2 (2)	3 (1.5)
1 person in 5,000	5 (4.9)	5 (2.4)
1 person in 10,000	60 (59.5)	123 (59.4)
I do not know	24 (23.8)	40 (19.3)
What is the estimated number of rare diseases?		
100–500	19 (18.8)	25 (12.1)
1,000–2,000	10 (9.9)	16 (7.7)
3,000–5,000	2 (2)	11 (5.3)
**6,000**–**8,000**	**5 (4.9)**	**15 (7.3)**
9,000–10,000	1 (1)	2 (1)
Over 10,000	4 (4)	16 (7.7)
I do not know	60 (59.4)	122 (58.9)
In what age group do rare diseases most frequently appear?		
Newborns	8 (7.9)	22 (10.6)
**Children**	**18 (17.8)**	**29 (14)**
Adolescents	3 (3)	2 (1)
Adults	1 (1)	7 (3.4)
They are present in all age groups equally	57 (56.4)	129 (62.3)
I do not know	14 (13.9)	18 (8.7)
How many people suffer from rare diseases worldwide?		
10–15,000,000	12 (11.9)	27 (13)
50–75,000,000	9 (8.9)	10 (4.8)
100–150,000,000	4 (4)	10 (4.8)
200–250,000,000	1 (1)	4 (1.9)
**300**–**350,000,000**	**1 (1)**	**9 (4.4)**
Over 500,000,000	3 (3)	9 (4.4)
I do not know	71 (70.2)	138 (66.7)
How many people suffer from rare diseases in Kazakhstan?		
250–500	9 (8.9)	17 (8.2)
5–7,500	9 (8.9)	24 (11.6)
25–40,000	7 (6.9)	11 (5.3)
50–75,000	4 (4)	7 (3.4)
150–250,000	2 (2)	13 (6.2)
500,000	4 (4)	3 (1.5)
**1**–**1,500,000**	**2 (2)**	**5 (2.4)**
Over 2,500,000	0 (0)	3 (1.5)
I do not know	64 (63.3)	124 (59.9)
What is the most common cause of rare diseases?		
Infectious and bacterial	4 (4)	9 (4.3)
**Genetic**	**65 (54.3)**	**123 (59.4)**
Autoimmune	14 (13.8)	37 (17.9)
Mitochondrial	2 (2)	1 (0.5)
Environmental	3 (3)	11 (5.3)
I do not know	13 (12.9)	26 (12.6)
What percentage of rare diseases are of a genetic origin?		
5–10%	19 (18.8)	35 (16.9)
20%	12 (11.9)	32 (15.5)
50%	13 (12.9)	38 (18.4)
**80%**	**16 (15.8)**	**35 (16.9)**
100%	3 (3)	5 (2.4)
I do not know	38 (37.6)	62 (29.9)

*Correct answers are written in bold characters*.

From the presented list of 29 diseases (including 19 RDs), respondents chose those they considered to be rare (Table 3). In the group of physicians Duchenne muscular dystrophy, Pompe disease and Gaucher disease were most frequently recognized (40.6, 38.6, and 38.6% respectively), while students pointed to Niemann-Pick disease, Huntington disease and Pompe disease most often (44.0, 33.3, and 32.9% respectively). Only in the case of Niemann-Pick disease, students recognize it better than physicians. In all other cases, the results were similar, or the physicians indicated RDs better than students. Simultaneously, physicians from the study often classified Munchausen syndrome, halitosis and fibromyalgia as RDs, while students erroneously indicated to Munchausen syndrome, halitosis and Down syndrome.

**Table 3:** Which of the following diseases are considered to be rare in Kazakhstan?

**Diseases**	***N*** **(%)**		***p***
	**MD *n* = 101**	**Students** ***n* = 207**	
**Sickle cell anemia**	18 (17.8)	40 (19.3)	
**Cystic fibrosis**	26 (25.7)	53 (25.6)	
**Acromegaly**	7 (6.9)	23 (11.1)	
**Hemophilia**	21 (20.8)	35 (16.9)	
Down syndrome	6 (5.9)	31 (15)	0.02
**Niemann-Pick disease**	30 (29.7)	91 (44)	0.02
Halitosis	16 (15.8)	39 (18.8)	
Glaucoma	3 (3)	5 (2.4)	
**Progeria**	37 (36.6)	43 (20.8)	<0.01
**Neurofibromatosis**	16 (15.8)	28 (13.5)	
**Craniodiaphyseal dysplasia**	17 (16.8)	31 (15)	
Cerebral palsy	4 (4)	23 (11.1)	0.04
Fibromyalgia	9 (8.9)	7 (3.4)	0.04
**Huntington disease**	32 (31.7)	69 (33.3)	
**Duchenne muscular dystrophy**	41 (40.6)	59 (28.5)	0.03
Acquired immunodeficiency syndrome	5 (5)	12 (5.8)	
Munchausen syndrome	24 (23.8)	68 (32.9)	
**Mucopolysaccharidoses**	13 (12.9)	24 (11.6)	
**Achondroplasia**	19 (18.8)	16 (7.7)	<0.01
**Galactosemia**	8 (7.9)	7 (3.4)	
**Pompe disease**	39 (38.6)	68 (32.9)	
**Gaucher disease**	39 (38.6)	49 (23.7)	<0.01
**Fragile X syndrome**			
**Marfan syndrome**	23 (22.8)	49 (23.7)	
Schizophrenia	3 (3)	14 (6.8)	
Alzheimer's disease	3 (3)	27 (13)	<0.01
**Osteogenesis imperfecta**	27 (26.7)	14 (6.8)	<0.001
**Phenylketonuria** Lactose intolerance	23 (22.8) 8 (7.9)	35 (16.9) 17 (8.2)	

*Correct answers are written in bold characters*.

Approximately 60% of respondents in both groups did not know whether Kazakhstan has a central register of RD patients (Table 4). Simultaneously, 33.8% of medical students and 40.65% of physicians falsely believed that there is a central register of RD patients in the country. Moreover, while very few respondents (4% of doctors and 7.2% of medical students) knew what percentage of RDs can be treated with drugs less than half knew that only some orphan drugs are reimbursed in RoK (48.5 and 43.5% respectively).

**Table 4:** Respondents knowledge about the healthcare system for RD patients in RoK.

	**N (%)**		***p***
	**MD *n* = 101**	**Students** ***n* = 207**	
Does Kazakhstan have a National Program for Rare Diseases?			ns
**Yes**	**31 (30.7)**	**62 (30)**	
No	10 (9.9)	21 (10.1)	
I do not know	60 (59.4)	124 (59.9)	
Is there a central register of RD patients in Kazakhstan?			ns
Yes	41 (40.6)	70 (33.8)	
**No**	**10 (9.9)**	**18 (8.7)**	
I do not know	50 (49.5)	119 (57.5)	
What percentage of rare disease can be treated with drugs?			ns
0%	5 (4.9)	4 (1.9)	
**5%**	**4 (4)**	**15 (7.2)**	
10%	2 (2)	17 (8.2)	
15%	9 (8.9)	19 (9.2)	
20%	10 (9.9)	37 (17.9)	
50%	19 (18.8)	18 (8.7)	
I do not know	52 (51.5)	97 (46.9)	
Are orphan drugs reimbursed in Kazakhstan?			ns
Yes	23 (22.8)	40 (19.3)	
**Yes, some**	**49 (48.5)**	**90 (43.5)**	
No	6 (5.9)	17 (8.2)	
I do not know	23 (22.8)	60 (29)	

*Correct answers are written in bold characters*.

Although more than 80% of the respondents in both groups agreed that RDs constitute a serious public health issue (Table 5), only 9.9% of physicians and 12.1% of students rated their knowledge about RDs as sufficient and the majority felt unprepared to care for RD (67.3 and 56.1% respectively). Interestingly, while almost half respondents declared having had classes about RDs during their studies, a statistically significant difference between the groups was found in primary source of knowledge on RDs: while only 11.9% physicians acknowledged past university classes, 25.6% of students believed university provided them with such knowledge. Moreover, while for most physicians the Internet, scientific symposia and literature was the prime source of information on RDs students pointed to the Internet, scientific literature and mandatory courses at the university. What was also significant, is that many physicians (16.8%) declared that they were not looking for information about RDs at all. At the same time, while in both groups the respondents believed that it is primarily family physicians (48.5 and 36.7%) and geneticists (33.7 and 44.9%) who should be uniquely educated and trained in RD, very few indicated to such specialists as pediatrician (24.8 and 23.7%), neurologist (15.8 and 16.4%) or psychiatrists (9.9 and 9.2%). Surprisingly, however, 64.4% of physicians and 72.5% of students believed that all physicians, regardless of their specialization, should possess such knowledge.

**Table 5:** Respondents' self-assessment of their knowledge about RDs.

	**N (%)**		***p***
	**MD *n* = 101**	**Students *n* = 207**	
Do RDs constitute a serious public health issue?			ns
Definitely yes	46 (45.5)	102 (49.3)	
Yes	39 (38.6)	69 (33.3)	
No	5 (5)	9 (4.3)	
Definitely not	3 (3)	3 (1.5)	
I do not know	8 (7.9)	24 (11.6)	
How would you rate your knowledge about rare diseases?			ns
Very good	4 (4)	8 (3.9)	
Fair enough	6 (5.9)	17 (8.2)	
So, so	39 (38.6)	94 (45.4)	
Insufficient	36 (35.6)	69 (33.3)	
Very poor	16 (15.8)	19 (9.2)	
Do you feel prepared for caring for a patient with a rare disease?			ns
Definitely yes	9 (8.9)	14 (6.7)	
Rather yes	12 (11.9)	43 (20.8)	
Rather not	47 (46.5)	84 (40.6)	
Definitely not	21 (20.8)	32 (15.5)	
I do not know	12 (11.9)	34 (16.4)	
Would you like to broaden your knowledge about rare diseases?			ns
Yes	86 (85.1)	183 (88.4)	
No	5 (5)	11 (5.3)	
I do not know	10 (9.9)	13 (6.3)	
Do you think that there should be a mandatory course on rare diseases in the medical curricula?			ns
Definitely yes	52 (51.5)	87 (42)	
Rather yes	38 (37.6)	96 (46.4)	
Rather not	3 (3)	7 (3.4)	
Definitely not	2 (2)	4 (1.9)	
I do not know	8 (7.9)	24 (11.6)	
Did you / do you have any classes about rare disease during your studies?			ns
Yes	48 (47.5)	98 (47.4)	
No	42 (41.6)	87 (42)	
I do not know	11 (10.9)	22 (10.6)	
Where do you / did you get your knowledge about rare diseases from?			
Mandatory courses at the university	12 (11.9)	53 (25.6)	<0.01
Facultative courses at the university	11 (10.9)	34 (16.4)	ns
Scientific literature and research	31 (30.7)	56 (27.1)	ns
Scientific conferences, symposia	20 (19.8)	15 (7.2)	<0.01
Internet	52 (51.5)	140 (67.6)	<0.01
Other	8 (7.9)	13 (6.3)	ns
I do not search for such information	17 (16.8)	12 (5.8)	<0.01
Which physicians should be uniquely trained in RDs?			
Family physician	49 (48.5)	76 (36.7)	0.05
Pediatrician	25 (24.8)	49 (23.7)	ns
Neurologist	16 (15.8)	34 (16.4)	ns
Geneticist	34 (33.7)	93 (44.9)	ns
Psychiatrist	10 (9.9)	19 (9.2)	ns
Immunologist	19 (18.8)	50 (24.2)	ns
Other	16 (15.8)	34 (16.4)	ns
Every physician regardless of their specialization	65 (64.4)	150 (72.5)	ns

## Discussion

Over the last few years the problem of rare diseases has been actively discussed in Kazakhstan. Consequently, both the government and medical authorities have recognized that RDs constitute an important public health and social issue (2, 12, 13, 15, 31). However, even though the Republic of Kazakhstan gained its independence in 1991 up till 2007 its medical education has continued to develop under the conditions of the methodological basis, structure and content established while being the republic of the Soviet Union (32). Moreover, Kazakhstan inherited the teacher-centered discipline-based system of medical education aimed to educate very large numbers of students in a few medical schools. Additionally, the traditional system was characterized by the development of a common schedule for all academic disciplines during the semester and the session. The development of individual study plans for each individual student was not envisaged. The notions of “elective disciplines,” “tutor,” “advisor,” “registrar's office” were absent in the traditional educational system, as well as the opportunity to choose disciplines, teachers and individual educational trajectory (4, 32–34).

Indeed, it was not until 2006 when the Ministry of Healthcare launched the reform of medical education and developed the new curriculum for all medical schools. For the very first time it introduced such new elements as integrated systems-based learning, early clinical contact, structured teaching of communication skills and promoted the development of student-centered methods of instruction (33). Hence, new State Compulsory Education Standards (the SCES) were introduced for medical specialties in higher education institutions (HEIs), but students of General Medicine and Dentistry specialties were trained according to the linear (traditional) system (35). Consequently, in order to obtain MD degree in general medicine, pediatrics or dentistry in Kazakhstan, there is currently a Bachelor's degree and internship program (5+1). This six-year of compulsory training is followed by residency which enables the future doctor to choose a specialization (36, 37).

However, although according to the SCES and the Model Curriculum the content of medical education program includes several blocks of disciplines, including general education disciplines (primarily socio-humanitarian disciplines), basic disciplines (natural sciences) and principal (clinical) disciplines), some of which are compulsory while other are elective (35, 37) there is no separate compulsory subject for RDs. Nevertheless, almost all diseases included in the List of Orphan Diseases and Medicines For Their Treatment can be found on the lists of Model residency curricula depending on the specialty. Additionally, examination of the Catalog of elective disciplines for residency of Medical University of Astana between 2017 and 2020 showed three elective disciplines on rare diseases in the profile tracks. They were “Orphan diseases in the Republic of Kazakhstan” and “Orphan diseases in pediatric practice” for Pediatrics specialty and “Orphan lung diseases and accumulation diseases” for Pulmonology specialty. Thus, apart from some limitations in the study programs HEIs can regulate and organize part of the content of their education programs.

It should be also stressed that the route of a RD patient in Kazakhstan is as follows: a patient with a suspected disease is referred by general practitioner or pediatrician to the regional level (to the Regional or City Children's Hospital), where one is examined by a regional coordinator and narrow profile specialists. At this level, the patient undergoes initial diagnosis, follow-up care, follow-up examinations and rehabilitation. After that, final verification of the diagnosis, primary therapy, monitoring within the specified timeframe and correction of therapy is required. This takes place at the national level: in Research Institutes, Science Centers, University Clinics or Rare Disease Coordination Centers, including Scientific Center of Pediatrics and Children's Surgery for children and Research Institute of Cardiology and Internal Medicine of the Ministry of Healthcare of the RoK for adults (37).

All in all, while Kazakhstani healthcare system suffers from the imbalance and low-skilled personnel it seems that one of the most important need in reference to RDs is that the standards of medical education require a revision both at the undergraduate and postgraduate level (33, 34). However, similar problems were found in other countries in the region. For example, a recent study from Russia showed that the main problems that prevent the full development of a public health strategy for RDs in the Russian Federation are insufficient organization of the process, lack of knowledge, lack of diagnostic expertise, lack of information on point prevalence and distribution of RDs by medical areas. It also showed that medical doctors, and those in primary care in particular, lack knowledge and experience on the diagnosis, treatment and rehabilitation of RD patients (31). Also in Turkey national policy for rare diseases and orphan drugs requires urgent updating as Turkish RD patients struggle with lack of knowledge and experience from healthcare practitioners, lack of specialist physicians and difficulties in patients' treatment and follow-up which in turn result in late or misdiagnosis, delayed access to appropriate treatment centers and inadequate number of orphan drugs (38). Finally, although awareness of RDs in many countries of Southeast Asia, including the Philippines, Singapore, Malaysia, Indonesia, Vietnam, and Thailand, is grounded on patient support and advocacy group they also suffer from insufficient number of genetic specialists, lack clinical RD expertise and possess only a few institutional centers that offer specific services to treat RDs. Consequently, clinical expertise and patient management for such diseases in these countries also need to be improved (39).

Moreover, this research supports findings from other countries that have shown that both medical students and healthcare professionals lack training and experience on RDs and that many problems of RD patients result from their negative experiences with healthcare system (40–44). For example, several Polish studies conducted among nursing, physiotherapy and medical students and practicing physicians and nurses showed that they possess insufficient knowledge about RDs and do not feel prepared to care for such patients (28, 29, 45–49). Also research conducted in Spain (50, 51) and Belgium (52) showed that most physicians do not possess adequate knowledge on RDs and rarely use Orphanet or other reliable sources on the Internet. Finally, recent surveys from China indicated the importance of improving awareness of RDs among physicians as only 5.3% were “moderately or well aware of” rare diseases (53).

Consequently, 73% of RD patients in China were misdiagnosed and waited an average more than 4 years for the right diagnosis and reported visiting three hospitals before receiving it. Additionally, 67% were diagnosed outside their home city and had to traveled an average of 562 km (54). Similarly, while an average time Polish patients suffering from Huntington disease had to wait for diagnosis was 10 years (55), for Australian children with RD it was up to 18 years (56). Finally, a research conducted among American and British caregivers showed that RD patients waited approximately 3 years before receiving a correct diagnosis (57–59).

### Limitations

Although to best of our knowledge this is the first study on the knowledge and awareness of RDs among medical students and practicing physicians in the Republic of Kazakhstan, it has some limitations. First, because responses from only one medical university in the Kazakhstan were analyzed the study has a local dimension. Consequently, it would be desirable to compare the findings from other medical universities in the country. Second, because the response rate was not very high the results represent solely the opinions of those who agreed to participate in the study and cannot be generalized for the entire population of medical students enrolled in the West Kazakhstan Marat Ospanov Medical University or medical doctors practicing in Aktobe, Kazakhstan. Thus, in order to clarify the issues of education for RD a more in-depth study is required. Third, non-random sampling is another limitation as it prevented an analysis of the socio-demographic, structural and socio-cultural background of the issues discussed in our research. However, some advantages of this study should also be acknowledged. Most importantly, as there is a scarcity of previous work on the topic it gives some highlight on the knowledge of Kazakhstani medical students and medical doctors about RD. Moreover, we believe that because this is a pilot study, it may stimulate further research on the topic and provoke discussion on the educational needs related RDs.

### Conclusions

Even though in the past 10 years Kazakhstan has made some progress in the management of RDs, still they are neglected by medical education in the country. Consequently, neither medial students nor physicians are offered special training on rare diseases and there is an urgent need to revise standards of medical education at the undergraduate, internship and residency level. Moreover, while the government should define clear guidelines regarding list of knowledge and skills in the field of RDs that future healthcare professionals should acquire during their studies, physicians (in training) should be offered opportunity to acquire experience in managing RD and interdisciplinary observation teams comprising of various specialists should be organized. Simultaneously, as the Internet seems the main source of information on RDs, special web pages with reliable information on RDs should also be organized.

Additionally, to ensure that RDs are adequately coded and traceable in Kazakhstani health information systems, the country should use experiences from other countries, including France, Germany, Spain or Poland, that alongside the existing coding system for RDs have decided to utilize the Orphanet nomenclature (ORPHAcodes), a comprehensive classification and coding system for RDs developed by the international consortium Orphanet, with cross-references to the ICD-10. Thus, RoK should put more effort to steer, maintain and promote the adoption of Orphacodes (60). Moreover, specially designed free mobile apps that would help physicians to diagnose RDs, find links to resources like disease information and connect RD patients, parents and caregivers with their physician would be also desirable. Finally, in order to support the decision process and overcome barriers that affect the diagnostic odyssey machine learning and artificial intelligence can be used for automatic surveillance for RD patients (61, 62).

Simultaneously, there are several other areas in the field of RDs that require substantial development. First, while RDs should be further be recognized and an important public health issue providing medical care for patients with specific RDs should be prioritized. Second, regional, national, and global health programs for the most common RDs need to be developed.

Third, standards for RD treatment guidelines should be developed. Fourth, as Kazakhstan suffers from inadequate number of medical geneticists in a number of regions, special courses on both clinical genetics and RDs should be organized, as it would foster the timely diagnosis, prevention of some RDs and referral of RD patients to specialized medical centers. Fifth, because many physicians do not possess knowledge on orphan drugs also pharmaceutical education on orphan drugs should be included in the medical curricula. Moreover, as pharmacists are credible sources of information on orphan drugs they should be also included in education and prevention programs on RDs (41, 43). Sixth, a system of follow-up facilities for RD patients must be developed. One possible way to achieve that is the development of telemedicine and telepharmacy services which can give RD patients the opportunity to continue treatment, to be followed-up by family physicians and specialists and to receive further recommendations about the therapy (63, 64). Additionally, a psychological, social and economic assistance and support for both the patients and their parents/caregivers needs to be developed (65). Seventh, an effective unit of the Ministry of Healthcare for RDs and orphan drugs should work actively to establish the necessary examinations, inspections, and relevant legislation. Eighth, because challenges faced by RD patients in RoK are similar to other countries in the region, including Russian Federation or China, the country should establish closer collaboration with other neighbor countries. Finally, further development and implementation of HTA for RD patients registries is required.

## Data Availability Statement

The raw data supporting the conclusions of this article will be made available by the authors, without undue reservation.

## Ethics Statement

The studies involving human participants were reviewed and approved by West Kazakhstan Marat Ospanov Medical University (Conclusion No 6, protocol No 2 of 02/18/2021). Written informed consent for participation was not required for this study in accordance with the national legislation and the institutional requirements.

## Author Contributions

DW supervised conceptualization of the study and performed the statistical analyses. JD designed of the research questionnaire. AM and KB collected the data. All authors conducted the literature search and analyses, had full access to all of the study data, discussed the results of the questionnaire, assisted in the interpretation of the data, wrote the original draft of the manuscript, critically revised and edited the various drafts of the manuscript and approved its final version before submission. All authors read and approved the final manuscript and contributed equally to the study.

## Conflict of Interest

The authors declare that the research was conducted in the absence of any commercial or financial relationships that could be construed as a potential conflict of interest.

## Publisher's Note

All claims expressed in this article are solely those of the authors and do not necessarily represent those of their affiliated organizations, or those of the publisher, the editors and the reviewers. Any product that may be evaluated in this article, or claim that may be made by its manufacturer, is not guaranteed or endorsed by the publisher.

## Footnotes

^1^https://www.rarediseaseday.org/countries/kazakhstan/ (accessed January 5, 2022).

## Abbreviations

RD: Rare disease
EU: The European Union
CEE: Central Eastern Europe
ICD-10: International Statistical Classification of Diseases and Related Health Problems
MDs: Medical doctors
RoK: the Republic of Kazakhstan
PHC: Primary healthcare
MoH: the Ministry of Healthcare
HTA: health technology assessment
OMP: orphan medicinal products
SCES: State Compulsory Education Standards
HEI: higher education institution.

## References

[1] Schieppati A Henter JI Daina E Aperia A. Why rare diseases are an important medical and social issue. Lancet. (2008) 371:2039–41. 10.1016/S0140-6736(08)60872-7 18555915

[2] Rodwell C Aymé S. Evolution of national and European policies in the field of rare diseases and their impact over the past five years. Orphanet J Rare Dis. (2014) 9:P13. 10.1186/1750-1172-9-S1-P13 PMC3942519 24580800

[3] Rodwell C Aymé S. Rare disease policies to improve care for patients in Europe. Biochim Biophys Acta. (2015) 1852(10 Pt B):2329–35. 10.1016/j.bbadis.2015.02.008 25725454

[4] Kawalec P Sagan A Pilc A. The correlation between HTA recommendations and reimbursement status of orphan drugs in Europe. Orphanet J Rare Dis. (2016) 11:122. 10.1186/s13023-016-0501-4 27600717 PMC5012088

[5] Zelei T Molnárm MJ Szegedi M Kaló Z. Systematic review on the evaluation criteria of orphan medicines in Central and Eastern European countries. Orphanet J Rare Dis. (2016) 11:72. 10.1186/s13023-016-0455-6 27259284 PMC4893267

[6] Dharssi S Wong-Rieger D Harold M Terry S. Review of 11 national policies for rare diseases in the context of key patient needs. Orphanet J Rare Dis. (2017) 12:63. 10.1186/s13023-017-0618-0 28359278 PMC5374691

[7] Moliner AM Waligora J. The European Union policy in the field of rare diseases. Adv Exp Med Biol. (2017) 1031:561–87. 10.1007/978-3-319-67144-4_30 29214592

[8] Khosla N Valdez R. A compilation of national plans, policies and government actions for rare diseases in 23 countries. Intractable Rare Dis Res. (2018) 7:213–22. 10.5582/irdr.2018.01085 30560012 PMC6290840

[9] Kolasa K Zwolinski KM Zah V Zoltán K Lewandowski T. Revealed preferences towards the appraisal of orphan drugs in Poland - multi criteria decision analysis. Orphanet J Rare Dis. (2018) 13:67. 10.1186/s13023-018-0803-9 29703227 PMC5922020

[10] Szegedi M Zelei T Arickx F Bucsics A Cohn-Zanchetta E. The European challenges of funding orphan medicinal products. Orphanet J Rare Dis. (2018) 13:184. 10.1186/s13023-018-0927-y 30396361 PMC6219168

[11] Montserrat A Taruscio D. Policies and actions to tackle rare diseases at European level. Ann Ist Super Sanità. (2019) 55:296–304. 10.4415/ANN_19_03_17 31553326

[12] Czech M Baran-Kooiker A Atikeler K Demirtshyan M Gaitova K Holownia-Voloskova M. A Review of Rare Disease Policies and Orphan Drug Reimbursement Systems in 12 Eurasian Countries. Front Public Health. (2020) 7:416. 10.3389/fpubh.2019.00416 32117845 PMC6997877

[13] Hedley V Murray H Rodwell C Aymé S. Overview Report on the State of the Art of Rare Disease Activities in Europe 2018 Version RD-ACTION WP6 Output. (2018). Available online at: https://ec.europa.eu/research/participants/documents/downloadPublic?documentIds=080166e5bd4653ff&appId=PPGMS (accessed January 5, 2022).

[14] Mayrides M Ruiz de Castilla E Szelepski S. A civil society view of rare disease public policy in six Latin American countries. Orphanet J Rare Dis. (2020) 15:60. 10.1186/s13023-020-1314-z 32106873 PMC7047392

[15] Pejcic AV Iskrov G Raycheva R Stefanov R Jakovljevic MM. Transposition and implementation of EU rare disease policy in Eastern Europe. Expert Rev Pharmacoecon Outcomes Res. (2017) 17:557–66. 10.1080/14737167.2017.1388741 28975845

[16] Aбдилова ГК Боранбаева PЗ Тилки-Шиманска A Плехановна ТA. Клинико-диагностическая характеристка детей с болезнью Гоше в Pеспублике Казахстан. Педиатрия и детская хирургия. (2018) 4:16–24.

[17] Pоль регионального координатора по редким заболеваниям в реализации Дорожной карты ≪Внедрение новыхстандартовдиагностикиилеченияредкихболезнейудетейвPК≫,. Научный центр педиатрии и детской хирургии MЗ PК Aлматы (2017). Available online at: http://www.gorbol2.akmol.kz/public/uploads/_RAZDELI/pacientam/orf_bol/2.pdf (accessed January 5, 2022)

[18] Приложение по диагностике редких заболеваний разработали в Казахстане (2020). Available online at: https://liter.kz/prilozhenie-po-diagnostike-redkih-zabolevanij-razrabotali-v-kazahstane/ (accessed January 5, 2022).

[19] Перечень зарегистрированных диссертаций PhD защищенных в Pеспублике Казахстан данные Национального научного портала. Available online at: https://nauka.kz/page.php?page_id=916&lang=1&parent_id=911 (accessed January 5, 2022).

[20] Приложение, *21* к Приказу *M*инистра здравоохранения *P*К №*30* от *31* января *2019* года ≪Дорожная картаповнедрениюновыхстандартовдиагностикиилеченияредкихзаболеванийудетейвPК на 2019-2020 гг,.≫. Available online at: http://www.rcrz.kz/files/DK2019/21.%20ДК%20орфан%20рус.pdf (accessed January 5, 2022).

[21] Pаспоряжение Премьер-Mинистра Pеспублики Казахстан от 17 августа 2020 года №2020 ≪Об утверждении Дорожной карты по совершенствованию оказания комплексной помощи детям с ограниченными возможностями в Pеспублике Казахстан на 2021-2023 годы≫. Available online at: https://adilet.zan.kz/rus/docs/R2000000112 (accessed January 5, 2022).

[22] Приказ Mинистра здравоохранения Pеспублики Казахстан от 12 ноября 2020 года № ҚP ДСM - 188/2020 ≪Об утвержденииправилразработкиипересмотраклиническихпротоколов≫. Available online at: https://adilet.zan.kz/rus/docs/V2000021637 (accessed January 5, 2022).

[23] Приказ Mинистра здравоохранения Pеспублики Казахстан от 16 октября 2020 года № ҚP ДСM-135/2020 ≪Об утвержденииправилформированияперечняорфанныхзаболеванийилекарственныхсредствдляихлечения≫. Available online at: https://adilet.zan.kz/rus/docs/V2000021454 (accessed January 5, 2022).

[24] Совещание по проблемным вопросам лекарственного обеспечения детей с орфанными заболеваниями и эпилепсией в MЗ PК от 08 июня 2021 года (2021). Available online at: https://bala-ombudsman.kz/i-snova-ob-orfannyh-zabolevaniyah/ (accessed January 5, 2022).

[25] Bektur C. Development of Hta in Kazakhstan. Value Health. (2016) 19:A807–918. 10.1016/j.jval.2016.08.760

[26] Black N Martineau F Manacorda T. Diagnostic Odyssey for Rare Diseases: Exploration of Potential Indicators. London: Policy Innovation Research Unit, LSHTM (2015).

[27] Carmichael N Tsipis J Windmueller G Mandel L Estrella E. “Is it going to hurt? “: the impact of the diagnostic odyssey on children and their families. J Genet Couns. (2015) 24:325–35. 10.1007/s10897-014-9773-9 25277096

[28] Domaradzki J Walkowiak D. Medical students' knowledge and opinions about rare diseases: a case study from Poland. Intractable Rare Dis Res. (2019) 8:252–9. 10.5582/irdr.2019.01099 31890452 PMC6929592

[29] Walkowiak D Domaradzki J. Needs assessment study of rare diseases education for nurses and nursing students in Poland. Orphanet J Rare Dis. (2020) 15:167. 10.1186/s13023-020-01432-6 32600383 PMC7322909

[30] Eurostat. Brancato G Macchia S Murgia M Signore M Simeoni G. The handbook of recommended practices for questionnaire development and testing in the European Statistical System (2005). Available online at: https://ec.europa.eu/eurostat/ramon/statmanuals/files/Handbook_of_Practices_for_Quest.pdf (accessed January 4, 2022).

[31] Zinchenko RA Ginter EK Marakhonov AV Petrova NV Kadyshev VV Vasilyeva TP. Epidemiology of rare hereditary diseases in the European part of Russia: Point and cumulative prevalence. Front Genet. (2021) 12:678957. 10.3389/fgene.2021.678957 34527017 PMC8435741

[32] Досмагамбетова PС Pиклефс ИM Pиклефс ВП Букеева AС. и др. Особенности медицинского образования в Казахстане. Mедицинское образование и профессиональное развитие. (2014) 4:75–85. Available online at: https://cyberleninka.ru/article/n/osobennosti-meditsinskogo-obrazovaniya-v-kazahstane (accessed December 30, 2021).

[33] Riklefs V Abakassova G Bukeyeva A Kaliyeva S Serik B Muratova A. Transforming medical education in Kazakhstan: Successful case of internationalization from Karaganda State Medical University. Med Teach. (2018) 40:481–7. 10.1080/0142159X.2018.1441989 29527966

[34] Abdrakhmanova A Koikov V Khandillayeva B. Problems of Health and Medical Education of the Republic of Kazakhstan and their solutions. J Clin Med Kazakhstan. (2015) 1:66–8.26639732

[35] Опыт преподавания дисциплины ≪Внутренние болезни≫ помодульно-линейной технологии Кудабаева Х.И., Зеленцова С.Ф., Базаргалиев Е.Ш., Турдалина A.К. Mедицинский журнал Западного Казахстана. (2012) 2:38–9.

[36] Приказ *M*инистра здравоохранения *P*еспублики Казахстан от *21* февраля *2020* года № Қ*P* ДС*M-12/2020* ≪О внесении изменений в приказ исполняющего обязанностей *M*инистра здравоохранения и социального развития *P*еспублики Казахстан от *31* июля *2015* года № *647* ≪Об утверждениигосударственныхобщеобязательныхстандартовитиповыхпрофессиональныхучебныхпрограммпомедицинскимифармацевтическимспециальностям≫. Available online at: https://adilet.zan.kz/rus/docs/V2000020071 (accessed January 5, 2022).

[37] Кодекс PК. О здоровье народа и системе здравоохранения от *7* июля *2020* года № *360-VI* З*P*К. Available online at: https://adilet.zan.kz/rus/docs/K2000000360 (accessed January 5, 2022).

[38] Koçkaya G Atalay S Oguzhan G Kurnaz M Ökçün S Sar Gedik Ç. Analysis of patient access to orphan drugs in Turkey. Orphanet J Rare Dis. (2021) 16:68. 10.1186/s13023-021-01718-3 33549137 PMC7868010

[39] Shafie AA Chaiyakunapruk N Supian A Lim J Zafra M Hassali MA. State of rare disease management in Southeast Asia. Orphanet J Rare Dis. (2016) 11:107. 10.1186/s13023-016-0460-9 27484654 PMC4969672

[40] Byrne PC. Training medical students on rare disorders. Orphanet J Rare Dis. (2012) 7:A15. 10.1186/1750-1172-7-S2-A15

[41] Krajnović D Arsić J Jocić D Milošević Georgiev A Tasić L Marinković V. Evaluation of pharmacists' knowledge and attitudes regarding rare disease and orphan drugs. Acta Med Mediterr. (2013) 52:23–32. 10.5633/amm.2013.0204

[42] Wolyniak MJ Bemis LT Prunuske AJ. Improving medical students' knowledge of genetic disease. a review of current and emerging pedagogical practice. Adv Med Educ Pract. (2015) 6:597–607. 10.2147/AMEP.S73644 26604852 PMC4629947

[43] Alam T Hameed A Naveed S Sharif N. Rare diseases: awareness amongst pharmacy students in Karachi, Pakistan. J Pharm Sci. (2016) 4:95–101.

[44] Medić B Divac N Stopić N Savić-Vujović K Glišić A Cerovac N. The attitudes of medical students towards rare diseases: a cross-sectional study. Vojnosanit Pregl. (2016) 73:703–13. 10.2298/VSP150326094M 29328568

[45] Kopeć G Podolec P. Establishing a curriculum on rare diseases for medical students. J Rare Cardiovascular Dis. (2015) 2:74–6. 10.20418/jrcd.vol2no3.19417515664

[46] Jonas K Waligóra M Hołda M Sulicka-Grodzicka J Strach M Podolec P. Knowledge of rare diseases among health care students – the effect of targeted education. Przegl Epidemiol. (2017) 71:80–9. 28742309

[47] Domaradzki J Walkowiak D. Knowledge and attitudes of future healthcare professionals towards rare diseases. Front Genet. (2021) 12:639610. 10.3389/fgene.2021.639610 34122502 PMC8194301

[48] Walkowiak D Domaradzki J. Are rare diseases overlooked by medical education? Awareness of rare diseases among physicians in Poland: an explanatory study. Orphanet J Rare Dis. (2021) 16:400. 10.1186/s13023-021-02023-9 34583737 PMC8479904

[49] Bokayeva K Miraleyeva A Walkowiak D. Rare diseases – a challenge for the medical world. JMS. (2021) 90:e503. 10.20883/medical.e503

[50] Avellaneda Fernández A Pérez Martín A Pombo Allés G Gutiérrez Delgado E Izquierdo Martínez M. nombre del Grupo de Trabajo de Enfermedades Raras de Semergen. Perception of rare diseases by the primary care physicians. Semergen. (2012) 38:421–31. 10.1016/j.semerg.2012.02.011 23021574

[51] Ramalle-Gómara E Domínguez-Garrido E Gómez-Eguílaz M Marzo-Sola ME Ramón-Trapero JL. Gil-de-Gómez J. Education and information needs for physicians about rare diseases in Spain. Orphanet J Rare Dis. (2020) 15:18. 10.1186/s13023-019-1285-0 31952528 PMC6969468

[52] Vandeborne L van Overbeeke E Dooms M De Beleyr B Huys I. Information needs of physicians regarding the diagnosis of rare diseases: a questionnaire-based study in Belgium. Orphanet J Rare Dis. (2019) 14:99. 10.1186/s13023-019-1075-8 31054581 PMC6500578

[53] Li X Zhang X Zhang S Lu Z Zhang J Zhou J. Rare disease awareness and perspectives of physicians in China: a questionnaire-based study. Orphanet J Rare Dis. (2021) 16:171. 10.1186/s13023-021-01788-3 33849615 PMC8042908

[54] Yan X He S Dong D. Determining how far an adult rare disease patient needs to travel for a definitive diagnosis: A cross-sectional examination of the 2018 National Rare Disease Survey in China. Int J Environ Res Public Health. (2020) 17:1757. 10.3390/ijerph17051757 32182694 PMC7084251

[55] Domaradzki J. Family caregivers' experiences with healthcare services - a case of Huntington disease. Psychiatr Pol. (2016) 50:375–91. 10.12740/PP/59103 27288682

[56] Anderson M Elliott EJ Zurynski YA. Australian families living with rare disease: experiences of diagnosis, health services use and needs for psychosocial support. Orphanet J Rare Dis. (2013) 8:22. 10.1186/1750-1172-8-22 23398775 PMC3599672

[57] Eurordis. The Voice of 12,000 Patients. Experiences and Expectations of Rare Disease Patients on Diagnosis and Care in Europe. (2009). Available online at: https://www.eurordis.org/IMG/pdf/voice_12000_patients/EURORDISCARE_FULLBOOKr.pdf (accessed December 14, 2021).

[58] Limb L Nutt S Sen A. Experiences of Rare Diseases: An Insight from Patients Families. (2010). Available online at: https://www.raredisease.org.uk/media/1594/rduk-family-report.pdf (accessed December 14, 2021).

[59] Shire Human Genetic Technologies. Rare Disease Impact Report: Insights From Patients and the Medical Community (2013). Available online at: https://globalgenes.org/wp-content/uploads/2013/04/ShireReport-1.pdf (accessed December 14, 2021).

[60] Kole A Hedley V. Recommendations from the Rare 2030 Foresight Study: The Future of Rare Diseases starts Today. (2021). Available online at: http://download2.eurordis.org/rare2030/Rare2030_recommendations.pdf (accessed March 15, 2022).

[61] De La Vega FM Chowdhury S Moore B Frise E McCarthy J. Artificial intelligence enables comprehensive genome interpretation and nomination of candidate diagnoses for rare genetic diseases. Genome Med. (2021) 13:153. 10.1186/s13073-021-00965-0 34645491 PMC8515723

[62] Decherchi S Pedrini E Mordenti M Cavalli A Sangiorgi L. Opportunities and challenges for machine learning in rare diseases. Front Med. (2021) 8:747612. 10.3389/fmed.2021.747612 34676229 PMC8523988

[63] Smith M Alexander E. Marcinkute R Dan D Rawson M Banka S. Telemedicine strategy of the European Reference Network ITHACA for the diagnosis and management of patients with rare developmental disorders. Orphanet J Rare Dis. (2020) 15:103. 10.1186/s13023-020-1349-1 32334637 PMC7183125

[64] Lochmüller H Ramirez AN Kakkis E. Disease monitoring programs of rare genetic diseases: transparent data sharing between academic and commercial stakeholders. Orphanet J Rare Dis 2021;16:141. 10.1186/s13023-021-01687-7 33743771 PMC7980582

[65] Cardinali P Migliorini L Rania N. The Caregiving Experiences of Fathers and Mothers of Children With Rare Diseases in Italy: Challenges and Social Support Perceptions. Front Psychol. (2019) 10:1780. 10.3389/fpsyg.2019.01780 31428029 PMC6690318

